# Progress in Bacteriology

**Published:** 1900-07-28

**Authors:** 


### Progress in Bacteriology.

**Tuberculosis.**—The committee of the Pathological Society[1] appointed to consider the nomenclature of the conditions sometimes described as pseudo-tuberculosis, report that the following conditions have been confused with true tuberculous nodules: Blastomycosis, streptotrichosis, aspergillosis, protozoal infection, and a number of bacterial infections caused by bacilli and cocci of various species. Some of these last-named organisms closely resemble Koch's bacillus, but differ in staining reactions and pathogenicity. Marzinowsky[2] gives the following method of distinguishing between the tubercle, leprosy, and smegma bacilli: Stain sections or films in a mixture of one part carbol-fuchsin, two parts water, for three to eight minutes, wash in water, and stain in Löffler's methylene blue for from two to five minutes. By this method the B. tuberculosis hominis is unstained, the B. tuberculosis avium is stained red, and is only decolourised by prolonged treatment with alcohol. The B. lepræ is also stained red, but is readily decolorised by alcohol; the B. smegmæ is, if allowed to remain for the full time in the blue, first stained violet and then blue.

**The Thermal Death Point of Bacillus Tuberculus.**—Sternberg[3] found the infectivity of sputum to be destroyed by heating to 60 deg. C. for 10 minutes. Tersin suggests 70 deg. C. for 10 minutes, Graucher and Ledoux-Lebard 70 deg. C. for five minutes, but in all cases dried material was much more resistant. Theobald Smith[4] investigated this matter with especial reference to milk. He found tubercle bacilli destroyed by exposure to 60 deg. C. for 20 minutes, provided no scum was allowed to form. Galtier,[5] on the other hand, states that milk heated for from 5 to 20 minutes at 75 deg. C. may still be infective. The agglutination reaction as a test of tuberculosis is not entirely reliable. Knopf[6] tested ten undoubted cases of pulmonary tuberculosis; four reacted positively, two slightly, one was doubtful, and one negative. One case of miliary tuberculosis gave only a doubtful reaction, as also did cases of chronic hemiplegia and alcoholic neuritis. No reaction was obtained from cases of tertiary syphilis and acute rheumatism.

**Enteric Fever.**—Horton-Smith[7] points out that no one of the ordinary tests employed for the detection of the typhoid bacillus can alone be relied upon. Many of the coli organisms, Gärtner's bacillus, and others very closely simulate it in nearly all its biological characters. The tests of most value are—(1) Morphology and immobility, the B. typhosus being long and exceptionally mobile; (2) flagellæ, more numerous in the real organism; (3) gelatin and agar slope culture; (4) formation of gas in 2 per cent. dextrose gelatin; (5) power of clotting and colouring litmus milk in one month's incubation; (6) formation of indol (one month); (7) agglutination test, using agar cultures, and dilutions of 1 in 1,000; (8) injection into guinea-pigs. The toxin of the typhoid bacillus is a feeble one, and is not diffused until the organism disintegrates. Remlinger has successfully produced the disease in rabbits. The wide distribution of the organism through the tissues of the human body shows us that the disease, far from being an intestinal disorder pure and simple, must be regarded as a modified form of septicæmia. For Widal's test an eighteen-hour broth culture should be used, dilution 1 in 20, the time of observation being one hour, thus eliminating the possibility of error due to Gärtner agglutins. The reaction may be found at the end of the first week, generally at the beginning of the second. In the great majority of cases it has disappeared by the end of a year. The agglutinating substance is an enzyme produced by the body cells; it plays no part in producing immunity. Antitoxins, if present, are formed only in small quantities in enteric fever. In typhoid bacilluria and cystitis urotropin, in doses of 30 grains daily, rapidly renders the urine sterile.

**Vaccine Lymphs.**[8]—The *Lancet* special commission on glycerinated calf lymphs reports as follows on the number and nature of the extraneous bacteria found: The admixture of glycerine with cultures of micro-organisms will cause the death of all organisms except certain forms of B. coli and spore-bearing bacteria in four weeks. Testing various lymphs, the very widest divergence of results was found, the average number of colonies varying from 2 to 6,882 on agar, and from 0 to 2,966 on gelatin. The extreme vitiation of certain samples may be due to lack of cleanliness in collecting, imperfect glycerination, or too brief a storage. Lymph should be stored for at least a month, and a bacteriological examination made of each batch of tubes. With glycerination the risks of inoculating syphilis or tuberculosis are reduced to a minimum.

**Notes.**—In cases of tropical abscess of the liver[9] the amœba coli is chiefly found about the walls of the abscess cavity, not in the contents. The discharge should, therefore, be examined after the abscess has been opened and drained for some days. If blood serum cultures from suspected cases of diphtheria be examined after six or seven hours' incubation the Klebs-Löffler bacilli, if present, may be detected. After the expiration of from twelve to sixteen hours growth is abundant. The Home Office is now prepared to make bacteriological examination of blood or tissues taken from suspected cases of anthrax arising in workshops or factories. Specimens must be sent to Dr. Whitelegge, the medical inspector. Levin,[10] who accompanied the Natthorst Arctic Expedition in 1898, remarks on the comparative absence of micro-organisms in this region. Testing samples of air (20,000 litres), he found no bacteria, whilst in sea water, snow, ice, and brown mud their occurrence was very rare. The intestinal contents of polar animals, birds, and fishes were nearly always sterile, so that the presence of bacteria cannot be regarded as essential to digestion. Mayer[11] describes an organism as the cause of intertrigo. It is a micrococcus, aërobic, growing well on all media, liquefying gelatine with the formation of a disagreeable odour like rotten cheese, staining with aniline dyes, and decolorised by Gram. The disease could be reproduced in animals in 48 hours. The coccus was speedily killed by a half per cent. solution of formalin.

[1] Brit. Med. Jour., May 20, 1899. [2] Centrab. f. Bak., xxv., 1899. [3] Public Health, April, 1900. [4] Jour. Exper. Med., Mar., 1899. [5] Soc. de Biolog., Feb. 3, 1900. [6] Med. Rec., Dec. 9, 1899. [7] Lancet, Mar. 24, 1900. [8] Ibid., April 21, 1900. [9] Trans. Jenner Instit., Series II. [10] Ann. Past. Inst., July, 1899. [11] New York Med. Jour., Dec. 16, 1899.

